# Household firearm ownership, firearm storage, and healthcare engagement

**DOI:** 10.1186/s12889-026-28498-4

**Published:** 2026-07-09

**Authors:** Alexander Testa, Shiven Patel, Annalyn S. DeMello, Mike Henson-Garcia, Dylan B. Jackson, Kyle T. Ganson, Sandra McKay, Jason M. Nagata

**Affiliations:** 1 Department of Management, Policy and Community Health, School of Public Health, University of Texas Health Science Center at Houston, Houston, TX USA; 2 University of Texas at Austin, Austin, TX USA; 3 Department of Research, Cizik School of Nursing, University of Texas Health Science Center at Houston, Houston, TX USA; 4 Institute for Firearm Injury Prevention, University of Michigan – Ann Arbor, Ann Arbor, MI USA; 5 Department of Population, Family and Reproductive Health, Johns Hopkins Bloomberg School of Public Health, Baltimore, MD USA; 6 Factor-Inwentash Faculty of Social Work, University of Toronto, Toronto, ON Canada; 7 Department of Pediatrics, McGovern Medical School, University of Texas Health Science Center at Houston, Houston, TX USA; 8 Division of Adolescent & Young Adult Medicine, Department of Pediatrics, University of California, San Francisco, San Francisco, CA USA

**Keywords:** Firearms, Firearm Storage, Healthcare Engagement, Injury Prevention

## Abstract

**Purpose:**

To assess whether (1) household firearm ownership and, among firearm-owning households, (2) household firearm storage are associated with healthcare engagement.

**Methods:**

Data were from the 2024 Behavioral Risk Factor Surveillance System from 19 US states (*N* = 145,720), including a subsample of respondents living in firearm-owning households (*n* = 54,125). Four indicators of healthcare engagement were assessed: health insurance status, having a personal healthcare provider, cost as a barrier to care, and time since last routine checkup. Multiply imputed, weighted multivariable logistic regression models were used to estimate associations between (1) household firearm ownership and (2) household firearm storage (unloaded, loaded and locked, and loaded and unlocked) and healthcare engagement, adjusting for age, sex, race/ethnicity, marital status, educational attainment, income, presence of a child in the home, military veteran, bad physical health days, urban-rural classification, and state of residence.

**Results:**

In covariate-adjusted models, household firearm ownership was associated with a higher odds of having a personal health care provider (adjusted odds ratio [aOR] = 1.12, 95% Confidence Interval [CI] = 1.03–1.21) compared with non-firearm-owning households. Household firearm ownership was not associated with any other healthcare engagement measure. Among firearm-owning households, storing firearms loaded and unlocked was associated with a lower odds lower odds of having health insurance (aOR = 0.65, 95% CI = 0.51–0.83), have a personal health care provider (aOR = 0.64, 95% CI = 0.54–0.76), and time since last routine checkup (aOR = 0.80, 95% CI = 0.69–0.92), compared to storing a firearm unloaded.

**Conclusions:**

These findings highlight the potential value of integrating firearm safety discussions into clinical visits and the need to develop strategies for reaching patients who store firearms unsecured and who engage less consistently with the healthcare system.

## Introduction

With approximately 300–400 million firearms in circulation, the United States (US) has the highest level of firearm ownership in the world [1, 2]. Importantly, the US also experiences a high level of firearm-related harms, including over 45,000 firearm deaths and nearly 100,000 non-fatal firearm injuries annually, which resulted in a cost of $493.2 billion in 2020 [3]. To address firearm-related harms, there has been increased attention focused on promoting firearm secure storage practices in homes (i.e., storing firearms locked, unloaded, and separate from ammunition) to reduce the risk of suicide and unintentional injury, particularly among children and adolescents [4, 5]. Even so, estimates suggest that 21% of firearm owners with a child in the home stored at least one firearm unlocked and loaded [6].

To promote greater firearm safety, organizations such as the American Medical Association (AMA) have encouraged physicians to counsel on firearm secure storage during patient visits [7]. The AMA recently launched a national secure-storage campaign (AgreeToAgree) aimed at normalizing clinician-led conversations about firearm safety and storage [8]. Notably, evidence suggests that clinician-led firearm safety counseling can increase the likelihood that firearms are stored locked and unloaded, particularly when counseling is paired with the provision of locking devices [4, 5]. However, limited research to date has examined the degree to if firearm owners visit healthcare providers, and whether firearm owners who engage with the healthcare system also engage in firearm secure storage. Paladhi et al. used the 2017 Behavioral Risk Factor Surveillance System (BRFSS) from six states (California, Idaho, Kansas, Oregon, Texas, and Utah) and found that individuals who did not live in a firearm-owning household were more likely to be uninsured, report no personal healthcare provider(s), and cite cost as a barrier to healthcare. Living in a household with a firearm stored loaded and unlocked – as compared to unloaded (either locked or unlocked) – was associated with a higher likelihood of not having a personal healthcare provider [9].

Beyond the Paladhi et al. study, no other research to the authors’ knowledge has assessed the relationship between (a) household firearm ownership, and (b) household firearm storage and healthcare engagement. This is a critical gap, considering that over the past decade, firearm ownership levels have increased, including millions of new gun owners since the COVID-19 pandemic [1, 10]. Alongside increasing levels of firearm ownership in recent years, there have also been declines in physician well visits among US adults [11]. Fittingly, the BRFSS has expanded the number of states that ask questions about household firearm ownership and storage, including 19 states in 2024. Thus, the current study addresses the following research questions: Are household firearm ownership status and household firearm storage practices associated with healthcare engagement, including (a) health insurance status, (b) having a personal healthcare provider(s), (c) cost as a barrier to healthcare, and (d) time since last routine checkup?

### Data

Data for the current study were from the 2024 BRFSS, an ongoing, state-based telephone survey conducted by state health departments in coordination with and supported by the Centers for Disease Control and Prevention. The BRFSS surveys noninstitutionalized US adults aged 18 years and older and is weighted to generate population-based estimates of health behaviors, preventive practices, and risk factors. While the core survey is administered in all 50 states and Washington, DC, participating sites may elect to include optional modules addressing specific topics [12]. The current study uses data from 19 states that implemented the optional firearm safety module in 2024, which asks about household firearm ownership and household firearm storage practices (see Appendix A for a list of states). Because BRFSS data are publicly accessible and contain no personally identifiable information, the study was exempt from Institutional Review Board review.

### Independent variable

Respondents living in states that participated in the firearm safety module received the following questions. First, *household firearm ownership* was asked via a single question “Are any firearms now kept in or around your home?” (yes or no). Next, *household firearm storage practices* were assessed among respondents who indicated household firearm ownership. Specifically, respondents were first asked, “Are any of these firearms now loaded?” (yes or no). Those who indicated the presence of loaded firearms were subsequently asked, “Are any of these firearms also unlocked?” (yes or no). Responses to these items were used to construct two variables. Household firearm ownership is a binary indicator (1 = yes; 0 = no) [13]. Among the subsample of households that reported owning firearms, we create an indicator of firearm storage: (a) unloaded, (b) loaded and locked, and (c) loaded and unlocked [9, 14].

### Dependent variable

Consistent with prior research,^9^ we create four binary dependent variables that capture healthcare engagement. *Health insurance* is a variable based on the question “What is the current primary source of your health care coverage?” Respondents are classified as 1 = insured or 0 = uninsured. *Personal healthcare provider* is based on the question “Do you have one person or a group of doctors that you think of as your personal health care provider?” (Yes, only one, Yes, more than one, or No). Responses were recoded as 1 = yes; 0 = no. *Cost as a barrier to care* is a binary variable based on the question: “Was there a time in the past 12 months when you needed to see a doctor but could not because you could not afford it? (1 = no; 0 = yes). *Time since last routine checkup* is based on the following question: “About how long has it been since you last visited a doctor for a routine checkup?” Response options include: (a) within the past year (anytime less than 12 months ago), (b) within the past 2 years (1 year but less than 2 years ago), (c) within the past 5 years (2 years but less than 5 years ago), (d) 5 or more years ago, and (e) never. Responses were recoded as 1 = within the past year or 0 = more than the past year.

### Control variables

Models were adjusted for demographic, socioeconomic, health-related, and contextual factors previously linked in the literature to firearm ownership and storage, which could confound the association among firearm ownership, storage, and healthcare engagement. Demographic variables included respondent age (18–24, 25–34, 35–44, 45–54, 55–64, or 65 years and older), sex (male or female), race/ethnicity (non-Hispanic White, non-Hispanic Black, non-Hispanic Multiracial, non-Hispanic other race, or Hispanic) given that firearm ownership and storage behaviors, as well as healthcare engagement vary by demographic groups [14–18]. Marital status (married, divorced or separated, widowed, never married, or part of an unmarried couple) is included to account for household composition, which may influence household firearm ownership and storage decisions, as well as health insurance and healthcare use patterns [14, 17, 19–21].

Socioeconomic indicators included educational attainment (less than high school, high school graduate, some college, or college graduate) and household income (<$25,000, $25,000 - $49,999, $50,000 - $74,999, $75,000 - $99,999, $100,000 - $149,999, or *≥* $150,000) as economic resources may influence access to firearms and firearm storage devices and healthcare engagement [14, 18, 22–25]. Additional covariates included whether a child lived in the home (yes/no), which consistently predicts firearm storage practices [14–17, 26]. Military veteran (yes or no) is included, given that firearm ownership and storage patterns may differ in veteran populations, given firearm training and exposure, and access to US Department of Veterans Affairs healthcare [27–30]. Models also accounted for the number of days in the past month a respondent reported poor physical health, given that health-related factors may impact health care use patterns and firearm-related behaviors [14, 24, 31, 32]. Finally, analyses controlled for urban-rural classification (urban or rural) and state of residence to account for geographic variations in firearm policy, storage behaviors, and healthcare engagement [14, 15, 33–35].

### Analytic approach

The analysis proceeds as follows. First, the summary statistics for the analytic sample were provided, including the full firearm safety module sample and the subsample of respondents in firearm-owning households who were asked about firearm storage. Next, a series of 8 total multiple logistic regression models was estimated to examine the relationships between (a) household firearm ownership and (b) household firearm storage on the four healthcare engagement variables, controlling for age, sex, race/ethnicity, marital status, educational attainment, income, presence of a child in the home, military veteran, bad physical health days, urban-rural classification, and state of residence.

To address missing data, multiple imputation by chained equations was used to generate 20 imputed datasets, and estimates were pooled across imputations using Rubin’s rules. Appendix B provides patterns of missing data across variables prior to imputation. All analyses accounted for the complex sampling design of the BRFSS by applying survey weights using the *svy* commands in Stata version 18 (StataCorp) [36]. Statistical significance was defined as *p* < .05 (two-tailed). Variance inflation factors (VIFs) were below 2.0 across models, indicating no significant issues with multicollinearity [37].

Following the main analysis, several supplemental analyses were conducted. First, because odds ratios can exaggerate relationships, [38] the results were reproduced using prevalence ratios. Next, supplemental analyses using listwise deletion yielded results were also conducted. Finally, given that firearm storage may differ between households with and without children, [14–17, 26] moderation analyses were conducted using interaction terms between the independent variables and the presence of a child in the home.

## Results

Among all respondents who participated in the firearm safety module (*n* = 145,720), 30.4% reported household firearm ownership, 92.0% reported having health insurance, 83.4% reported having a healthcare provider, 88.2% reported that cost was not a barrier to care, and 77.5% reported having their last routine checkup within the past year. Among those living in firearm-owning households (*n* = 54,125), 64.9% reported that firearms were stored unloaded, 18.4% reported storage as loaded and locked, and 16.7% reported storage as loaded and unlocked. Furthermore, in the firearm-owning households 95.2% reported having health insurance, 87.9% reported having a healthcare provider, 91.0% reported that cost was not a barrier to care, and 79.4% reported their last routine checkup was within the past year (see Table 1).

**Table 1:** Summary statistics from 2024 behavioral risk factor surveillance system

Variable	Participated in Firearm Safety Module (*N* = 145,720)			Firearm Owning Households (*N* = 54,125)		
	Unweighted *N*	Weighted %	95% CI	Unweighted *N*	Weighted %	95% CI
*Household Firearm Ownership*						
No	91,595	69.6%	69.1–70.1	—	—	—
Yes	54,125	30.4%	29.9–30.9	—	—	—
*Firearm Storage*						
Unloaded & Locked	—	—	—	36,355	64.9%	64.1–65.8
Loaded and Locked	—	—	—	8,391	18.4%	17.6–19.1
Loaded and Unlocked	—	—	—	9,379	16.7%	16.0–17.3
*Current Health Insurance*						
Uninsured	8,926	8.0%	7.7–8.3	1,993	4.8%	4.4–5.3
Insured	136,794	92.0%	91.7–92.3	52,132	95.2%	94.7–95.6
*Personal Healthcare Provider*						
No	17,802	16.6%	16.2–17.0	4,932	12.1%	11.5–12.7
Yes	127,918	83.4%	83.0–83.8	49,193	87.9%	87.3–88.5
*Cost as a Barrier to Care*						
Yes	14,221	11.8%	11.4–12.1	3,950	9.0%	8.4–9.5
No	131,499	88.2%	87.9–88.6	50,175	91.0%	90.5–91.6
*Time Since Last Routine Checkup*						
More than One Year	26,252	22.5%	22.0–22.9	8,844	20.6%	19.9–21.4
Within the Past Year	119,468	77.5%	77.1–78.0	45,281	79.4%	78.6–80.1
*Age*						
18–24	9,083	12.1%	11.8–12.5	2,486	9.5%	8.9–10.1
25–34	15,525	16.7%	16.3–17.1	4,522	14.2%	13.5–14.8
35–44	19,141	16.8%	16.4–17.2	6,507	15.8%	15.1–16.4
45–54	20,667	15.4%	15.0–15.8	8,050	16.7%	16.0–17.3
55–64	26,106	15.7%	15.3–16.1	10,810	18.1%	17.5–18.7
65+	55,198	23.3%	23.0–23.7	21,750	25.8%	25.1–26.4
*Sex*						
Female	77,031	50.9%	50.4–51.4	24,892	43.8%	42.9–44.7
Male	68,689	49.1%	48.6–49.6	29,233	56.2%	55.3–57.1
*Race/Ethnicity*						
Non-Hispanic White	105,958	54.2%	53.7–54.7	45,089	71.7%	70.8–72.6
Non-Hispanic Black	14,620	12.8%	12.5–13.1	3,937	11.0%	10.4–11.5
Non-Hispanic Other	7,028	10.5%	10.0–10.9	1,628	5.7%	5.1–6.3
Non-Hispanic Multiracial	3,172	2.7%	2.5–2.8	1,174	3.0%	2.6–3.4
Hispanic	14,942	19.8%	19.3–20.2	2,297	8.6%	7.9–9.3
*Marital Status*						
Married	72,963	49.5%	49.0–50.0	33,346	60.6%	59.7–61.4
Divorced/Separated	22,348	12.1%	11.8–12.4	6,770	10.7%	10.2–11.2
Widowed	16,017	6.6%	6.4–6.8	5,267	6.2%	5.8–6.5
Never Married	27,510	25.8%	25.4–26.3	6,685	18.0%	17.3–18.7
Member of an Unmarried Couple	6,882	5.9%	5.7–6.2	2,057	4.6%	4.2–4.9
*Educational Attainment*						
Less than High School	8,956	11.5%	11.1–11.9	1,958	6.4%	5.9–7.0
High School Graduate	36,460	26.3%	25.9–26.8	14,000	27.9%	27.1–28.7
Some College	38,286	29.8%	29.3–30.3	15,918	34.3%	33.5–35.2
College Graduate	62,018	32.3%	31.9–32.8	22,249	31.3%	30.6–32.1
*Income ($)*						
<25,000	22,712	15.5%	15.1–15.8	4,835	8.0%	7.5–8.4
25,000–49,999	37,051	24.4%	23.9–24.8	12,333	20.6%	19.9–21.3
50,000–74,999	23,650	14.9%	14.5–15.3	9,588	16.6%	15.9–17.4
75,000–99,999	19,714	13.1%	12.7–13.5	8,632	16.0%	15.3–16.7
100,000-149,999	21,170	14.5%	14.1–14.9	9,559	18.3%	17.5–19.0
>150,000	21,423	17.7%	17.2–18.2	9,178	20.5%	19.7–21.3
*Child in Home*						
None	109,454	67.1%	66.5–67.6	41,209	68.0%	67.2–68.8
1 or More	36,266	32.9%	32.4–33.5	12,916	32.0%	31.2–32.8
*Military Veteran*						
No	129,189	90.8%	90.5–91.1	45,027	84.7%	84.1–85.4
Yes	16,531	9.2%	8.9–9.5	9,098	15.3%	14.6–15.9
*Bad Physical Health Days*	—	5.6	5.5–5.7	—	5.2	5.0–5.4
*Urban-Rural Classification*						
Rural	18,613	5.4%	5.3–5.6	10,132	9.6%	9.2–10.0
Urban	127,107	94.6%	94.4–94.7	43,993	90.4%	90.0–90.8

Sample uses data from the 2024 BRFSS from the following states: Arizona, California, Illinois, Indiana, Louisiana, Maine, Maryland, Michigan, Minnesota, Mississippi, New Jersey, New Mexico, North Carolina, Oklahoma, Oregon, South Carolina, Virginia, West Virginia, Wyoming

The results of the multiple logistic regression are provided in Table 2. The findings indicate that household firearm ownership was associated with a higher odds of having a personal health care provider (adjusted odds ratio [aOR] = 1.12, 95% Confidence Interval [CI] = 1.03–1.21) compared with non-firearm-owning households. Household firearm ownership was not associated with any other healthcare engagement measure. Next, among firearm-owing households, the findings indicate that households storing firearms loaded and unlocked had a significantly lower odds of having health insurance (aOR = 0.65, 95% CI = 0.51–0.83), have a personal health care provider (aOR = 0.64, 95% CI = 0.54–0.76), and time since last routine checkup (aOR = 0.80, 95% CI = 0.69–0.92) compared to those storing firearms unloaded.

**Table 2:** Results of multiple logistic regression between household firearm ownership, household firearm storage, and healthcare engagement

Variable	Current Health Insurance	Personal Healthcare Provider(s)	Cost not a Barrier to Care	Time Since Last Routine Checkup (Past Year)
	aOR (95% CI)	aOR (95% CI)	aOR (95% CI)	aOR (95% CI)
Household Firearm Ownership *N* = 145,*720*	1.02 (0.90–1.15)	1.12** (1.03–1.21)	1.05 (0.95–1.16)	0.98 (0.91–1.05)
Firearm Storage *N* = 54,*125*				
Firearm Unloaded (ref.)	—	—	—	—
Loaded & Locked	0.86 (0.65–1.12)	0.93 (0.77–1.11)	0.86 (0.72–1.04)	0.95 (0.82–1.11)
Loaded & Unlocked	0.65** (0.51–0.83)	0.64*** (0.54–0.76)	0.87 (0.71–1.07)	0.80** (0.69–0.92)

Each column represents a separate logistic regression model for the exposure of (a) household firearm ownership, and (b) firearm storage. Adjusted models include the following control variables: age, race/ethnicity, biological sex, marital status, educational attainment, income, child in home, military veteran, poor physical health days, urban-rural classification, and state of residence. For firearm storage, the reference category is firearm unloaded; estimates are shown for loaded and locked and loaded and unlocked relative to that reference. Estimates are based on multiple imputation (20 imputations). Sample uses data from the 2024 BRFSS from the following states: Arizona, California, Illinois, Indiana, Louisiana, Maine, Maryland, Michigan, Minnesota, Mississippi, New Jersey, New Mexico, North Carolina, Oklahoma, Oregon, South Carolina, Virginia, West Virginia, Wyoming

*Abbreviations* *aOR* adjusted odds ratio, *CI* confidence interva

***p < .001, **p < .01, *p < .05

### Supplementary analyses

A series of supplementary analyses was conducted. First, an analysis of the results using modified Poisson regression, reporting prevalence ratios, yielded substantively similar findings, and the patterns of statistical significance were similar to those from logistic regression (see Appendix C). Supplemental analyses using listwise deletion yielded results similar to those from multiple imputation (see Appendix D). However, the relationship between household firearm ownership and personal healthcare provider was not statistically significant at the *p* < .05 level, although the coefficient remained substantially similar in magnitude. Finally, the results showed no significant differences in the overall findings in moderation analyses by the presence of a child in the home (see Appendix E).

## Discussion

Using data from 19 US states participating in the 2024 BRFSS, the current study updates and expands prior work by Paladhi et al.^9^ by using a greater number of states. Regarding household firearm ownership, Paladhi et al. found that those who were in non-firearm owning households were more likely to be uninsured, have no personal healthcare provider(s), and report cost as a barrier to care [9]. However, the current study found that household firearm ownership was associated with a higher odds of having a personal healthcare provider. Of note, Paladhi et al. adjusted for only age, sex, education, employment, children in the home, and state of residency. However, the current study included several additional covariates, including measures of respondents’ income and marital status that differed between respondents in and out of firearm-owning households, and are meaningful factors in understanding healthcare engagement.[20, 21, 25].

Next, regarding firearm storage practices and healthcare engagement, Paladhi et al. observed a significant association only between storing firearms loaded and unlocked and having no personal healthcare provider(s), relative to storing firearms unloaded. However, in the current study, the findings indicated that firearms stored loaded and unlocked, compared to unloaded, were associated with lower odds of currently having health care, having a personal healthcare provider(s), and having a last routine checkup within the past year. Overall, these findings are consistent with prior research showing that secure firearm storage is associated with uptake of preventive health behaviors (i.e., cholesterol screening, influenza immunization, physical activity, primary care, and seatbelt use) [39].

The findings suggest that secure storage counseling in healthcare clinics may be a feasible approach for reaching firearm-owning households. However, these strategies may miss a subgroup of firearm owners who are in households with unsecured firearm storage practices who are less engaged with the healthcare system. Additional research is needed to clarify the mechanisms linking unsecured firearm storage with lower healthcare engagement to inform more targeted prevention efforts. Future studies should also evaluate strategies and venues beyond healthcare systems, including partnerships with community-based settings such as firearm retailers, shooting ranges, and other trusted organizations that may better reach firearm-owning households who do not regularly engage with healthcare.

### Limitations and future directions

This study has limitations that should be considered when interpreting the findings. First, the observational design raises the possibility of residual or unmeasured confounding, and the self-report nature of the data on firearms and healthcare use patterns may result in reporting biases. Second, considering the BRFSS is cross-sectional, temporal ordering cannot be established. Future research using longitudinal data is needed to observe how healthcare engagement patterns evolve alongside changes to household firearm ownership and storage practices. Third, in the BRFSS, firearm ownership and storage are measured at the household level; thus, the respondent may not necessarily be the firearm owner or the individual who decides how firearms are stored. Fourth, it is also important to note that the odds ratios are slight in magnitude. Fifth, response rates vary across states (See Appendix A), and if they differ significantly by firearm ownership and storage practices, this may introduce bias into the study’s findings. Finally, although the data were drawn from 19 states, the results are not generalizable to the entire US population. In particular, several states with the highest (i.e., Alaska, Idaho, Montana) and lowest (i.e., Hawaii, Massachusetts, Rhode Island) levels of firearm ownership were not available in the current data [40]. Moving forward, additional data collection that expands information on firearm ownership and storage across states can help better understand how the observed relationships vary across states, and may be influenced by firearm and healthcare policy.

## Conclusion

The current study found that among firearm-owning households in 19 US states, those in firearm-owning households were more likely to report having a personal health care provider than those in non-firearm-owning households. However, firearm-owning households that stored firearms loaded and unlocked had a lower odds of having health insurance, a personal healthcare provider, and having had a routine checkup in the past year. These findings have implications for the reach and design of healthcare firearm storage interventions.

## Data Availability

The datasets generated and/or analyzed during the current study are publicly available. Data used in this study can be obtained at: https://www.cdc.gov/brfss/annual\_data/annual\_2024.html.

## Appendix A

### List of 19 states in analytic sample

**Table:**

State	Unweighted *N*	Weighted %	95% CI	BRFSS Response Rate	Average Gun Ownership by State (RAND)
Arizona	3,915	5.0%	4.9–5.2	42.1%	37%
California	8,382	25.8%	25.4–26.2	44.8%	18%
Illinois	11,980	8.3%	8.2–8.4	39.9%	24%
Indiana	13,050	4.5%	4.5–4.6	43.7%	40%
Louisiana	4,547	3.0%	2.9–3.0	38.6%	48%
Maine	12,144	1.0%	1.0–1.0	39.8%	45%
Maryland	9,432	8.2%	8.1–8.4	41.1%	21%
Michigan	11,419	6.7%	6.6–6.8	46.1%	35%
Minnesota	15,199	3.8%	3.7–3.8	41.5%	39%
Mississippi	2,986	1.9%	1.9–2.0	48.8%	50%
New Jersey	8,666	6.2%	6.1–6.3	35.7%	8%
New Mexico	3,393	1.4%	1.4–1.5	51.2%	39%
North Carolina	4,325	7.4%	7.2–7.5	45.6%	35%
Oklahoma	3,365	2.7%	2.6–2.7	56.2%	51%
Oregon	6,152	2.9%	2.8–2.9	43.9%	44%
South Carolina	9,485	3.7%	3.6–3.8	38.4%	43%
Virginia	6,860	5.8%	5.7–6.0	50.6%	37%
West Virginia	5,816	1.2%	1.2–1.2	55.3%	58%
Wyoming	4,604	0.4%	0.4–0.4	57.6%	59%

Note: Weighted percentages and 95% confidence intervals are based on survey-weighted, multiply-imputed estimates (20 imputations; *N* = 145,720). Unweighted N reflects the observed sample frequency

BRFSS response rates are derived from: https://www.cdc.gov/brfss/annual_data/2024/pdf/2024_ResponseRates_Table-508.pdf

Average household gun ownership by state is derived from: https://www.rand.org/research/gun-policy/gun-ownership.html

## Appendix B

### Percentage of missing data for study variables

**Table:**

Variable	*N* Missing	% Missing
Household Firearm Ownership	32,925	22.59%
Firearm Storage¹	1,316	3.08%
Current Health Insurance	5,757	3.95%
Personal Healthcare Provider	1,422	0.98%
Cost as a Barrier to Care	518	0.36%
Time Since Last Routine Checkup	1,676	1.15%
Age	0	0.00%
Sex	0	0.00%
Race/Ethnicity	2,796	1.92%
Marital Status	1,338	0.92%
Educational Attainment	738	0.51%
Income	27,648	18.97%
Child in Home	2,863	1.96%
Military Veteran	838	0.58%
Bad Physical Health Days	779	0.53%
Urban-Rural Classification	0	0.00%

¹ Missingness for firearm storage is calculated among firearm-owning respondents rather than the full sample

## Appendix C

### Results of modified poisson regression between household firearm ownership, household firearm storage, and healthcare engagement using prevalence ratios

**Table:**

Variable	Current Health Insurance	Personal Healthcare Provider(s)	Cost not a Barrier to Care	Time Since Last Routine Checkup (Past Year)
	aPR (95% CI)	aPR (95% CI)	aPR (95% CI)	aPR (95% CI)
Household Firearm Ownership *N* = 145,*720*	1.00 (1.00–1.01)	1.01* (1.00–1.02)	1.01 (0.99–1.01)	1.00 (0.98–1.01)
Firearm Storage *N* = 54,*125*				
Firearm Unloaded (ref.)	—	—	—	—
Loaded & Locked	0.99 (0.98–1.01)	0.99 (0.97–1.01)	0.99 (0.97–1.00)	0.99 (0.96–1.02)
Loaded & Unlocked	0.98* (0.97–1.00)	0.95*** (0.93–0.97)	0.99 (0.97–1.01)	0.96** (0.93–0.99)

Note: Each column represents a separate modified Poisson regression model (Poisson regression with survey-linearized robust variance) for the exposure of (a) household firearm ownership, and (b) firearm storage. Prevalence ratios (PR) are estimated as the exponentiated coefficients (incidence-rate ratios) from the Poisson models. Adjusted models include the following control variables: age, race/ethnicity, biological sex, marital status, educational attainment, income, child in home, military veteran, poor physical health days, urban-rural classification, and state of residence. For firearm storage, the reference category is firearm unloaded; estimates are shown for loaded and locked and loaded and unlocked relative to that reference. Estimates are based on multiple imputation (20 imputations). Sample uses data from the 2024 BRFSS from the following states: Arizona, California, Illinois, Indiana, Louisiana, Maine, Maryland, Michigan, Minnesota, Mississippi, New Jersey, New Mexico, North Carolina, Oklahoma, Oregon, South Carolina, Virginia, West Virginia, Wyoming

*Abbreviations* *aPR* adjusted prevalence ratio, *CI* confidence interval

****p* < .001, ***p* < .01, **p* < .05

## Appendix D

### Results of multiple logistic regression between household firearm ownership, household firearm storage, and healthcare engagement using listwise deletion

**Table:**

Variable	Current Health Insurance	Personal Healthcare Provider(s)	Cost not a Barrier to Care	Time Since Last Routine Checkup (Past Year)
	aOR (95% CI)	aOR (95% CI)	aOR (95% CI)	aOR (95% CI)
Household Firearm Ownership *N* = 90,*788*	0.93 (0.79–1.08)	1.10 (0.99–1.21)	1.06 (0.95–1.17)	0.97 (0.89–1.04)
Firearm Storage *N* = 34,*854*				
Firearm Unloaded (ref.)	—	—	—	—
Loaded & Locked	0.82 (0.62–1.09)	0.94 (0.78–1.14)	0.93 (0.76–1.13)	0.94 (0.81–1.09)
Loaded & Unlocked	0.65** (0.48–0.87)	0.60*** (0.49–0.73)	0.94 (0.76–1.17)	0.76*** (0.65–0.88)

Each column represents a separate logistic regression model for the exposure of (a) household firearm ownership, and (b) firearm storage. Adjusted models include the following control variables: age, race/ethnicity, biological sex, marital status, educational attainment, income, child in home, military veteran, poor physical health days, urban-rural classification, and state of residence. For firearm storage, the reference category is firearm unloaded; estimates are shown for loaded and locked and loaded and unlocked relative to that reference. Estimates are based on complete-case (listwise deletion) analysis. Sample uses data from the 2024 BRFSS from the following states: Arizona, California, Illinois, Indiana, Louisiana, Maine, Maryland, Michigan, Minnesota, Mississippi, New Jersey, New Mexico, North Carolina, Oklahoma, Oregon, South Carolina, Virginia, West Virginia, Wyoming

*Abbreviations* *aOR* adjusted odds ratio, *CI* confidence interval

****p* < .001, ***p* < .01, **p* < .05

## Appendix E

### Interaction between firearm ownership / storage and child in home on healthcare engagement using multiple imputation

**Table:**

Variable	Current Health Insurance	Personal Healthcare Provider(s)	Cost not a Barrier to Care	Time Since Last Routine Checkup (Past Year)
	aOR (95% CI)	aOR (95% CI)	aOR (95% CI)	aOR (95% CI)
Firearm Ownership × Child in Home *N* = 145,*720*				
Firearm Owner × 1 + Children	1.03 (0.86–1.29)	1.06 (0.91–1.25)	1.02 (0.85–1.22)	0.89 (0.79–1.01)
Firearm Storage × Child in Home *N* = 54,*125*				
Unloaded × Children (ref.)	—	—	—	—
Loaded & Locked × 1 + Children	1.05 (0.64–1.73)	0.74 (0.52–1.05)	1.11 (0.76–1.62)	0.89 (0.66–1.18)
Loaded & Unlocked × 1 + Children	0.88 (0.51–1.53)	0.91 (0.62–1.34)	0.72 (0.47–1.12)	0.98 (0.71–1.35)

Each column represents a separate survey-weighted logistic regression model containing the interaction between the firearm exposure and child in home (none vs. 1 or more). Cells report the adjusted odds ratio for the interaction (product) term only. Ownership models contrast firearm owners vs. non-owners; storage models are restricted to firearm owners with the unloaded category as the reference. Each model adjusts for age, race/ethnicity, biological sex, marital status, educational attainment, income, military veteran status, poor physical health days, urban-rural classification, and state of residence. Estimates are based on multiple imputation (20 imputations)

*Abbreviations aOR* adjusted odds ratio, *CI* confidence interval

**p* < .001, ***p* < .01, ****p* < .05
